# Hydrogels in cancer treatment: mapping the future of precision drug delivery

**DOI:** 10.3389/fimmu.2025.1607240

**Published:** 2025-07-08

**Authors:** Xiang Liu, Qiang Zhou, Yue Yang, Xin Wu, Jie Chen, Ruoqin Wang, Erhua Chen

**Affiliations:** ^1^ College of Traditional Chinese Medicine, Jiangsu College of Nursing, Huaian, Jiangsu, China; ^2^ Department of Clinical Pharmacy, Jinling Hospital, Medical school of Nanjing University, Nanjing, China

**Keywords:** hydrogels, cancer, drug delivery, bibliometrics, CiteSpace

## Abstract

**Background:**

Current primary tumor treatments include curative resection, chemotherapy, and radiotherapy. However, these conventional methods lack precise drug delivery. Hydrogels, adaptable to the biological characteristics of different tumors, offer potential as drug delivery systems and represent a significant area of research in tumor treatment. In this study, we aimed to conduct a bibliometric analysis to reveal the current progress and future prospects of hydrogels for drug delivery in cancer.

**Methods:**

Publications concerning hydrogels in tumor drug delivery were retrieved from the Web of Science Core Collection (WoSCC) database. Data regarding countries/regions, institutions, journals, authors, and document types were collected. Bibliometric analysis and network visualization were performed using CiteSpace, HisCite, VOSviewer, Alluvial Generator, and R software.

**Results:**

China, the United States, and India were the leading contributing countries. Of the 98 relevant categories, 94 experienced citation bursts between 2000 and 2024. The research team led by Professor Pourmadadi Mehrab demonstrated substantial influence in this field. The International Journal of Biological Macromolecules was the most prolific journal. The top three emergent categories originated in 2020 or later, are "Chemistry, Applied", "Engineering, Environmental", and "Biochemistry & Molecular Biology". "Designing hydrogels for controlled drug delivery" was the most highly cited article. Recent emergent keywords included immunotherapy, immunogenic cell death, carboxymethyl cellulose, and antibacterial. Key concept alluvial flow visualization revealed six terms: peritoneal carcinomatosis, iron oxide nanoparticles, drug delivery, release kinetics, carbon dots, and pathway. Nano-composite hydrogels, immunotherapy, quercetin, pancreatic cancer, and oral cancer exhibited recent activity within the cited article timeline, suggesting these areas as potential future research hotspots.

**Conclusion:**

This bibliometric analysis identifies future research directions within the developing field of hydrogels for drug delivery in cancer. This study provides recommendations and directions for the development of hydrogels as tumor drug delivery systems.

## 1 Introduction

Cancer remains a significant threat to human health (1). Current tumor treatments include surgery, radiotherapy, chemotherapy, and traditional Chinese medicine, with chemotherapy being a common clinical approach (2, 3). While chemotherapy can improve patient survival, its lack of targeting can damage normal cells, leading to serious side effects (4). To improve chemotherapeutic efficacy, researchers have made significant progress in developing targeted drug delivery systems. These systems can deliver drugs directly to tumor cells through specific targeting mechanisms, thereby reducing side effects on healthy tissues and improving the effectiveness of the treatment (5). Advances in nanotechnology have provided new approaches to drug delivery systems. Nanocarriers can improve drug targeting and release efficiency through a variety of mechanisms (6). For example, pH-sensitive nanosystems can trigger drug release in the acidic tumor microenvironment, thereby improving therapeutic efficiency (7). These intelligent drug delivery systems can more effectively cross biological barriers for targeted intracellular drug delivery, leading to improved therapeutic outcomes. The drug delivery system combines low molecular weight cytotoxic drugs with nanocarriers, and this combination therapy can enhance the anti-tumor potency by promoting more uniform drug distribution within the tumor, thus improving therapeutic efficacy (8, 9). The structure of hydrogels includes polymeric materials with crosslinked 3D networks (10). Based on the raw materials used, hydrogels can be classified as either natural or synthetic (11). Hydrogel technology has experienced increased use in cancer treatment in recent years. Hydrogels, as drug delivery carriers, demonstrate considerable potential in tumor treatment. As semisolid drug delivery systems, they can effectively facilitate the transdermal transport of active drugs (12).

Injectable hydrogels offer minimally invasive *in vivo* delivery of chemotherapeutic drugs, representing a novel approach to cancer treatment (13). These hydrogels can reduce systemic side effects and improve therapeutic efficacy by increasing drug concentration at tumor sites. In addition, hydrogel nanocomposites can improve drug penetration within tumors, enhancing both penetration and residence time through a combination of active transcytosis and passive diffusion (14).

Bibliometrics employs statistical analysis of published works to evaluate the influence and development trends of scientific research through quantitative indicators. It is widely used across various fields to analyze research status, trends, hotspots, and collaborative patterns. CiteSpace, a tool focused on the visualization of scientific literature, identifies key documents and research frontiers by analyzing document co-citation networks. Researchers can use CiteSpace to construct scientific knowledge maps, facilitating a deeper understanding of the evolution of a given research field (15).

This study employed HisCite, CiteSpace, VOSviewer, and R software to generate a knowledge map. Using a bibliometric approach, we analyzed the knowledge framework, research hotspots, and trends in the development of hydrogels for drug delivery in cancer. This analysis provides further insights into hydrogels as drug delivery systems, especially in cancer treatment, hoping to provide new ideas and directions for targeted tumor delivery.

## 2 Methods

### 2.1 Data collection and strategies

**Figure 1:**
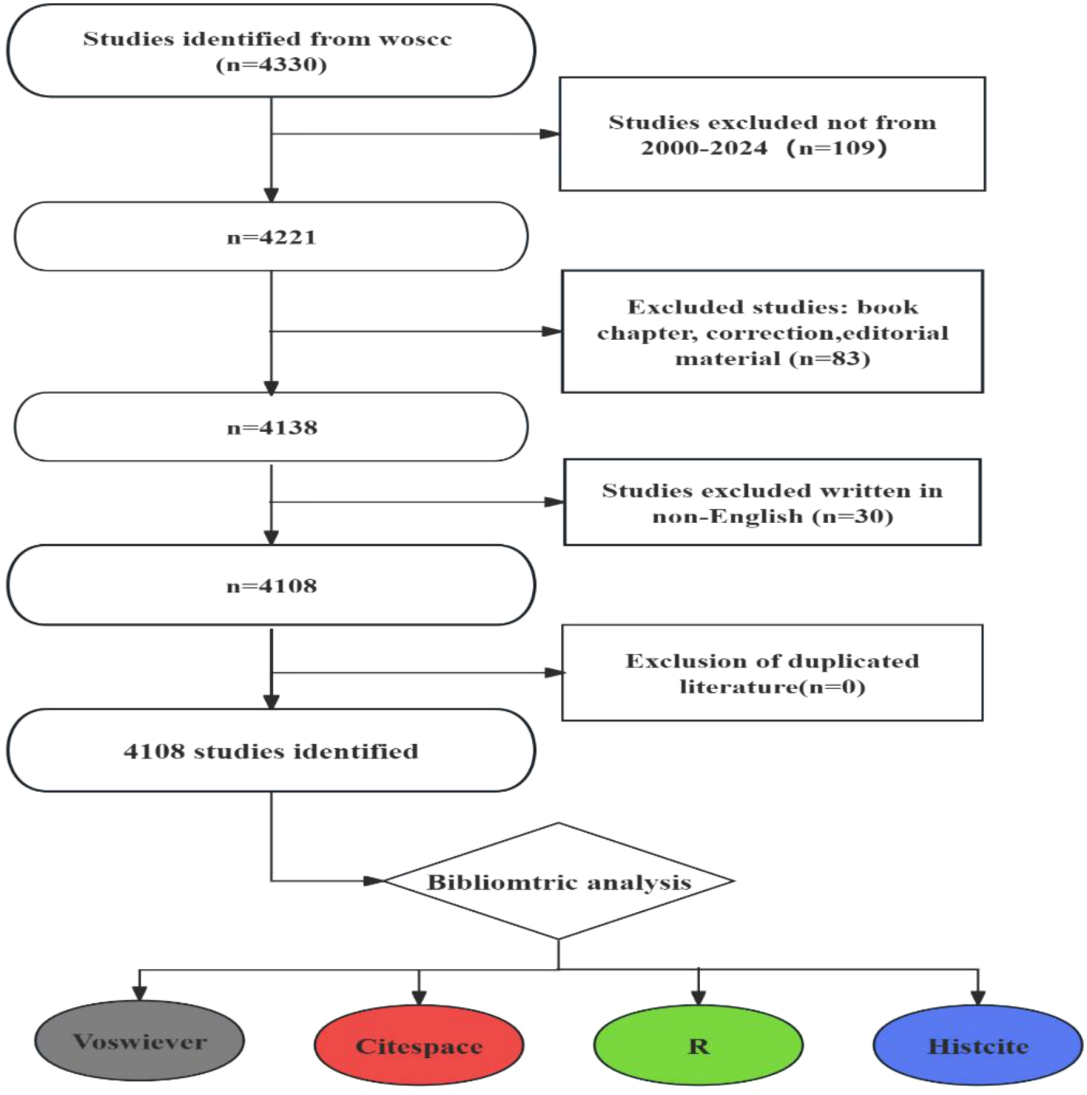
Flow diagram of search and study selection.

The Web of Science Core Collection (WoSCC), encompassing over 12,000 influential scholarly journals, is a widely recognized and comprehensive, multidisciplinary database offering citation analysis. For this study, we searched the WoSCC database for relevant literature published between 2000 and 2024. The search formula was ((TS=(“Tumor”) OR TS=(“Neoplasm”) OR TS=(“Tumors”) OR TS=(“Neoplasia”) OR TS=(“Neoplasias”) OR TS=(“Cancer”) OR TS=(“Cancers”) OR TS=(“Malignant Neoplasm”) OR TS=(“Malignancy”) OR TS=(“Malignancies”) OR TS=(“Malignant Neoplasms”) OR TS=(“Neoplasm, Malignant”) OR TS=(“Neoplasms, Malignant”) OR TS=(“Benign Neoplasms”) OR TS=(“Benign Neoplasm”) OR TS=(“Neoplasms, Benign”) OR TS=(“Neoplasm, Benign”))AND ((TS=(Hydrogels)) OR TS=(Hydrogel)) OR TS=(“Patterned Hydrogel”) AND (((((TS=(“Drug Delivery Systems”)) OR TS=(“Drug Delivery System”)) OR TS=(“Drug Targeting*”)) OR TS=(“Drug Carrier*”)) OR TS=(“Drug Carriers”)) OR TS=(“drug delivery”). We Retrieved records were downloaded as plain text files in “Full Record and Cited References” format. This data, comprising 4108 publications, was saved for subsequent analysis and designated as DATA (**Figure 1**). Finally, we collected information, including country, region, institution, journal, author, keywords, and key references. Data statistics were performed using Microsoft Excel 2019.

### 2.2 Data analysis

**Figure 2:**
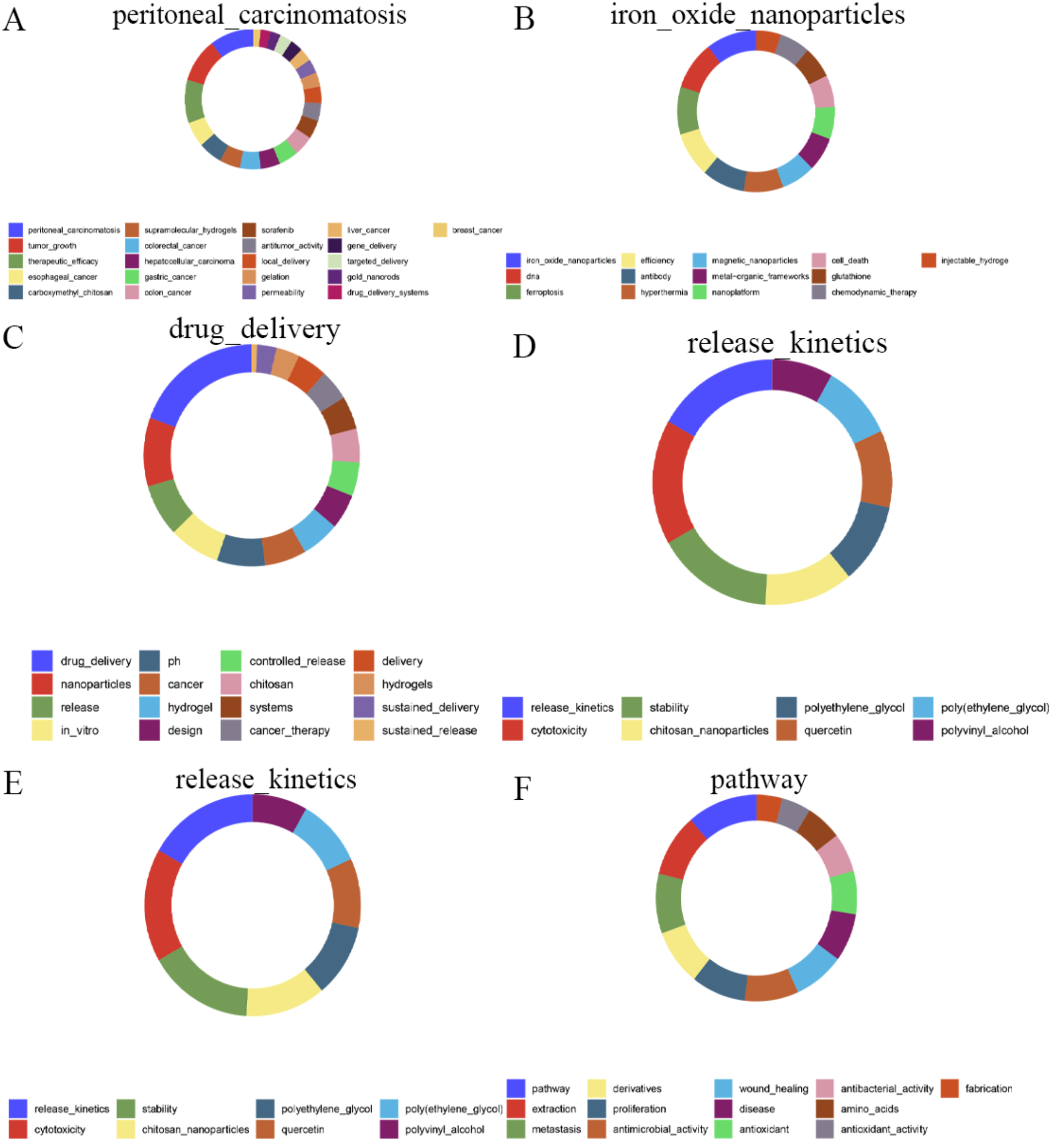
The keywords of top 5 modules in 2024. **(A)** Module 1. **(B)** Module 2. **(C)** Module 3. **(D)** Module 4. **(E)** Module 5. **(F)** Module 6.

CiteSpace uses distinct node and edge colors to differentiate the merged network, with each color representing a different year. This bibliometric analysis software provided three clustering algorithms based on titles, abstracts, and keywords. The resulting cluster maps reflect changes in conceptual clustering over time. Relevant data were imported into CiteSpace (version 6.2.R4). VOSviewer was used to visualize the author collaboration network. HistCite Pro (version 2.1) was used to plot numerical relationships, identify highly cited literature, and readily display the most influential publications. HistCite calculates both local citation scores (LCS) and global citation scores (GCS)) to identify authoritative and influential papers. Relevant data from 4108 research articles on hydrogels in cancer drug delivery were imported into HistCite Pro 2.1. Alluvial flow maps were generated using the Alluvial Flow Generator (http://www.mapequation.org/apps/AlluvialGenerator.html) to illustrate temporal patterns in evolving networks. The donut plot shown in **Figure 2** was generated using R (version 4.2.2) with the geom_bar function from the ggplot2 package (version 3.4.4).

## 3 Results

### 3.1 The historical overview of hydrogel research in cancer drug delivery

#### 3.1.1 Distribution of publications

The increase or decline in publications over time can reveal research interest in specific fields, providing quantitative insights for subject research and publication. In this study, a total of 4108 publications related to hydrogels in oncology drug delivery were retrieved, including 3110 research articles and 998 review articles, with 15,496 participating authors and 3293 participating institutions, published in 664 journals in 98 scientific categories.

**Figure 3:**
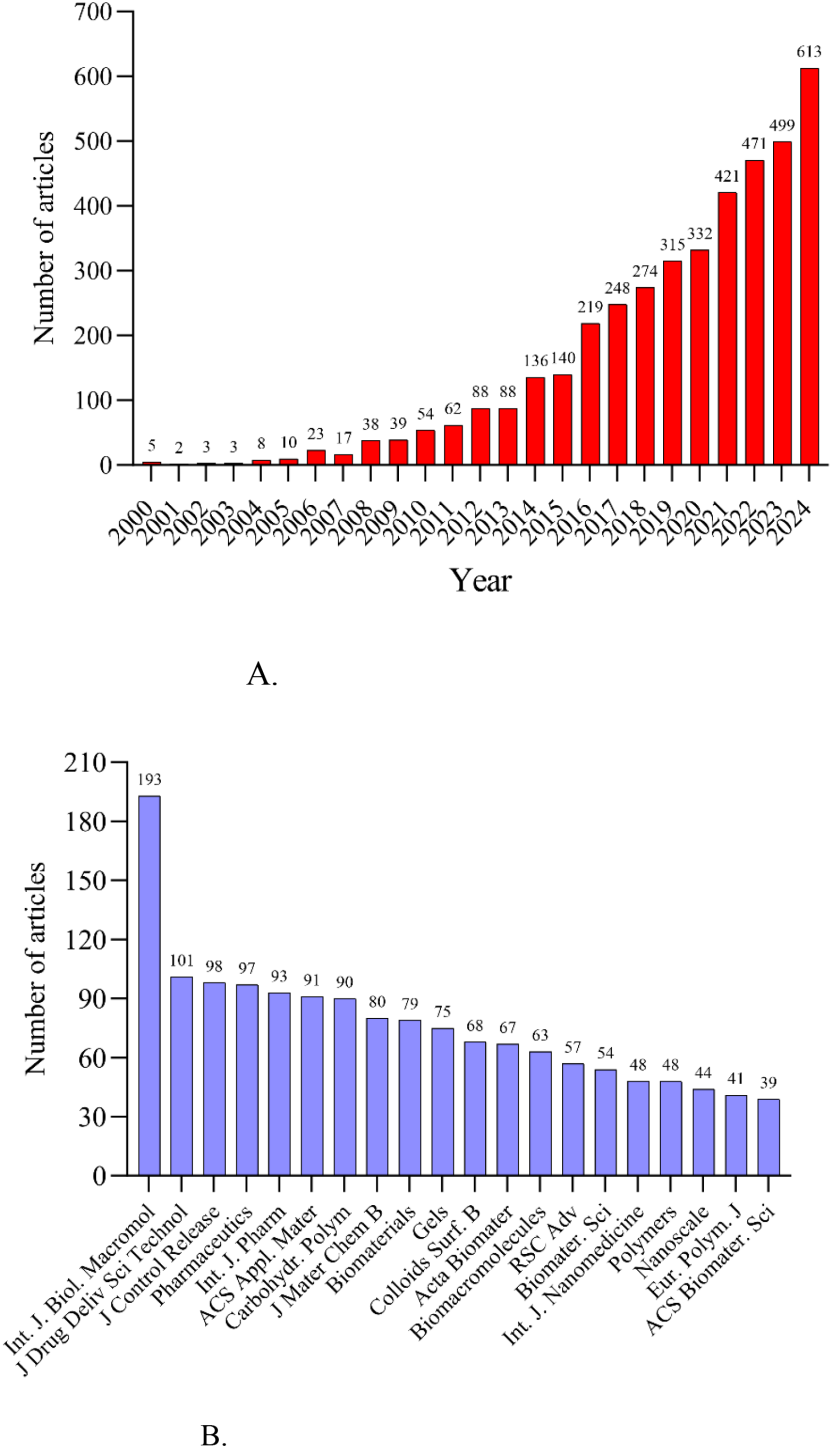
**(A)** Annual number of publications. **(B)** The top 20 journals by publication volume.

Annual research output is shown in **Figure 3A**. Five papers related to hydrogels in oncology drug delivery were published in 2000, and two in 2001. Publication numbers remained low from 2000 to 2007, followed by a rapid increase from 2008 to 2015. The growth rate further accelerated after 2016, peaking in 2024. The *International Journal of Biological Macromolecules* was the most prolific journal (n=193 publications), followed by the *Journal of Drug Delivery Science and Technology* (n=101) and the *Journal of Controlled Release* (n=98). **Figure 3B** and **Table 1** list the top 20 most productive journals, providing a useful reference for researchers considering manuscript submissions.

**Table 1:** The information of the top 20 journals.

Ranking	Journals	Impact factor
1	International journal of biological macromolecules	7.7
2	Journal of drug delivery science and technology	4.5
3	Journal of controlled release	10.5
4	Pharmaceutics	4.9
5	International journal of pharmaceutics	5.3
6	Acs applied materials & interfaces	8.5
7	Carbohydrate polymers	10.7
8	Journal of materials chemistry b	6.1
9	Biomaterials	12.8
10	Gels	5
11	Colloids and surfaces b-biointerfaces	5.4
12	Acta biomaterialia	9.4
13	Biomacromolecules	5.5
14	Rsc advances	3.9
15	Biomaterials science	5.8
16	International journal of nanomedicine	6.7
17	Polymers	4.7
18	Nanoscale	5.8
19	European polymer journal	5.8
20	Acs biomaterials science & engineering	5.5

#### 3.1.2 Co-citation network analysis of the evolution of hydrogel research in cancer drug delivery

**Figure 4:**
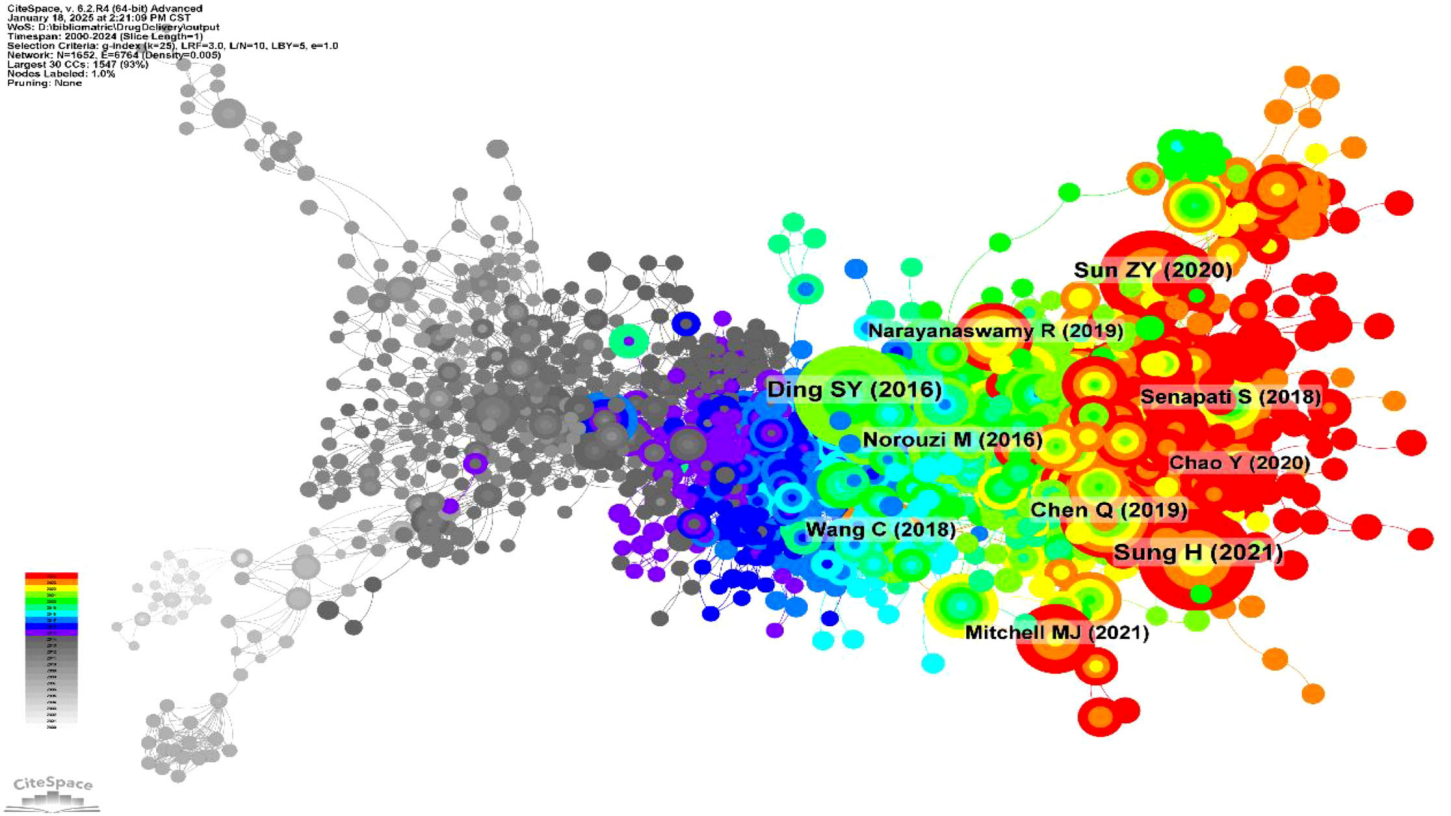
Knowledge map of co-citation literature.

A co-citation network was generated (**Figure 4**) to illustrate the relationships between publications in the field of hydrogels for oncology drug delivery from 2000 to 2024. The network comprised 1652 nodes and 6764 links, indicating extensive interconnections within this research area. Early literature (2000-2010), represented by densely interconnected gray nodes, forms the foundational “roots”, supporting the field’s subsequent growth. During the mid-period (2011-2017), blue-marked nodes became more dispersed, forming the main research branches. In the later period (2018-2024), nodes develop into tighter clusters, indicating concentration and differentiation within the field. Key publications driving this research, with their respective co-citation frequencies, included Sung H (2021) (16) (n=120), Ding SY (2016) (17) (n=116), Sun ZY (2020) (18) (n=99), Chen Q (2019) (19) (n=79), Norouzi M (2016) (20) (n=68), Wang C (2018) (21) (n=64), Senapati S (2018) (22) (n=61), Narayanaswamy R (2019) (23) (n=57), Mitchell MJ (2021) (24) (n=55), and Chao Y (2020) (25) (n=54). This concentration and differentiation of research clusters were further clarified in subsequent reference timeline plots. Citation histories of these research articles, visualized using HisCite Pro 2.1, are summarized in **Table 2**. The top three cited papers were “Injectable hydrogel-based drug delivery systems for local cancer therapy” (20), “Local drug delivery strategies for cancer treatment: Gels, nanoparticles, polymeric films, rods, and wafers” (26), and “Hydrogel-based controlled drug delivery for cancer treatment: A Review” (18). Larger nodes in the graph indicate greater citation frequency and, thus, greater importance of the referenced work.

**Table 2:** The information of the top 30 literature sorted by LCS score.

NO.	Article information	Journal	LCS	GCS
918	Injectable hydrogel-based drug delivery systems for local cancer therapy	DRUG DISCOV TODAY	163	409
307	Local drug delivery strategies for cancer treatment: Gels, nanoparticles, polymeric films, rods, and wafers	J CONTROL RELEASE	136	676
1835	Hydrogel-Based Controlled Drug Delivery for Cancer Treatment: A Review	MOL PHARMACEUT	103	371
1078	pH-responsive self-healing injectable hydrogel based on N-carboxyethyl chitosan for hepatocellular carcinoma therapy	ACTA BIOMATER	79	473
715	Engineered *in-situ* depot-forming hydrogels for intratumoral drug delivery	J CONTROL RELEASE	61	245
639	Nanocomposite Hydrogels: 3D Polymer-Nanoparticle Synergies for On-Demand Drug Delivery	ACS NANO	61	637
87	Injectable chitosan hydrogels for localised cancer therapy	J CONTROL RELEASE	61	139
933	Anticancer drug-loaded hydrogels as drug delivery systems for the local treatment of glioblastoma	J CONTROL RELEASE	59	197
1020	Designing Hydrogels for On-Demand Therapy	ACCOUNTS CHEM RES	53	1591
320	Designing Cell-Compatible Hydrogels for Biomedical Applications	SCIENCE	53	268
1133	Injectable and Self-Healing Thermosensitive Magnetic Hydrogel for Asynchronous Control Release of Doxorubicin and Docetaxel to Treat Triple-Negative Breast Cancer	ACS APPL MATER INTER	50	160
1591	Dual thermo-and pH-sensitive injectable hydrogels of chitosan/(poly(N-isopropylacrylamide-co-itaconic acid)) for doxorubicin delivery in breast cancer	INT J BIOL MACROMOL	49	353
530	Ultrasound-triggered disruption and self-healing of reversibly cross-linked hydrogels for drug delivery and enhanced chemotherapy	P NATL ACAD SCI USA	49	151
1057	Injectable, NIR/pH-Responsive Nanocomposite Hydrogel as Long-Acting Implant for Chemophotothermal Synergistic Cancer Therapy	ACS APPL MATER INTER	49	137
1241	Novel concept of the smart NIR-light-controlled drug release of black phosphorus nanostructure for cancer therapy	P NATL ACAD SCI USA	48	689
1525	pH-responsive injectable hydrogels with mucosal adhesiveness based on chitosan-grafted-dihydrocaffeic acid and oxidized pullulan for localized drug delivery	J COLLOID INTERF SCI	45	569
1143	Injectable nanomedicine hydrogel for local chemotherapy of glioblastoma after surgical resection	J CONTROL RELEASE	45	102
374	Doxorubicin: an update on anticancer molecular action, toxicity and novel drug delivery systems	J PHARM PHARMACOL	43	344
1312	Doxorubicin loaded carboxymethyl cellulose/graphene quantum dot nanocomposite hydrogel films as a potential anticancer drug delivery system	MAT SCI ENG C-MATER	43	1867
1153	Engineered Hydrogels in Cancer Therapy and Diagnosis	TRENDS BIOTECHNOL	42	222
869	Injectable and pH-Responsive Silk Nanofiber Hydrogels for Sustained Anticancer Drug Delivery	ACS APPL MATER INTER	42	167
774	Synergistic therapeutic effects of Schiff’s base cross-linked injectable hydrogels for local co-delivery of metformin and 5-fluorouracil in a mouse colon carcinoma model	BIOMATERIALS	41	129
1077	Thermo-sensitive polypeptide hydrogel for locally sequential delivery of two-pronged antitumor drugs	ACTA BIOMATER	40	165
714	Localized Co-delivery of Doxorubicin, Cisplatin, and Methotrexate by Thermosensitive Hydrogels for Enhanced Osteosarcoma Treatment	ACS APPL MATER INTER	37	95
1580	Paclitaxel-loaded pH responsive hydrogel based on self-assembled peptides for tumor targeting	BIOMATER SCI-UK	36	133
2096	Chitosan/carbon quantumdot/aptamer complex as a potential anticancer drug delivery system towards the release of 5-fluorouracil	INT J BIOL MACROMOL	36	417
302	Hyaluronic acid-based hydrogel for regional delivery of paclitaxel to intraperitoneal tumors	J CONTROL RELEASE	36	120
114	Advanced nanogel engineering for drug delivery	SOFT MATTER	36	86
662	Temozolomide-loaded photopolymerizable PEG-DMA-based hydrogel for the treatment of glioblastoma	J CONTROL RELEASE	36	95
1265	Injectable Hexapeptide Hydrogel for Localized Chemotherapy Prevents Breast Cancer Recurrence	ACS APPL MATER INTER	36	141

LCS, The total local citation score; GCS, The total global citation score. NO: The number of the literature in the database import into HisCite pro 2.1.

#### 3.1.3 Scientific cooperation

**Figure 5:**
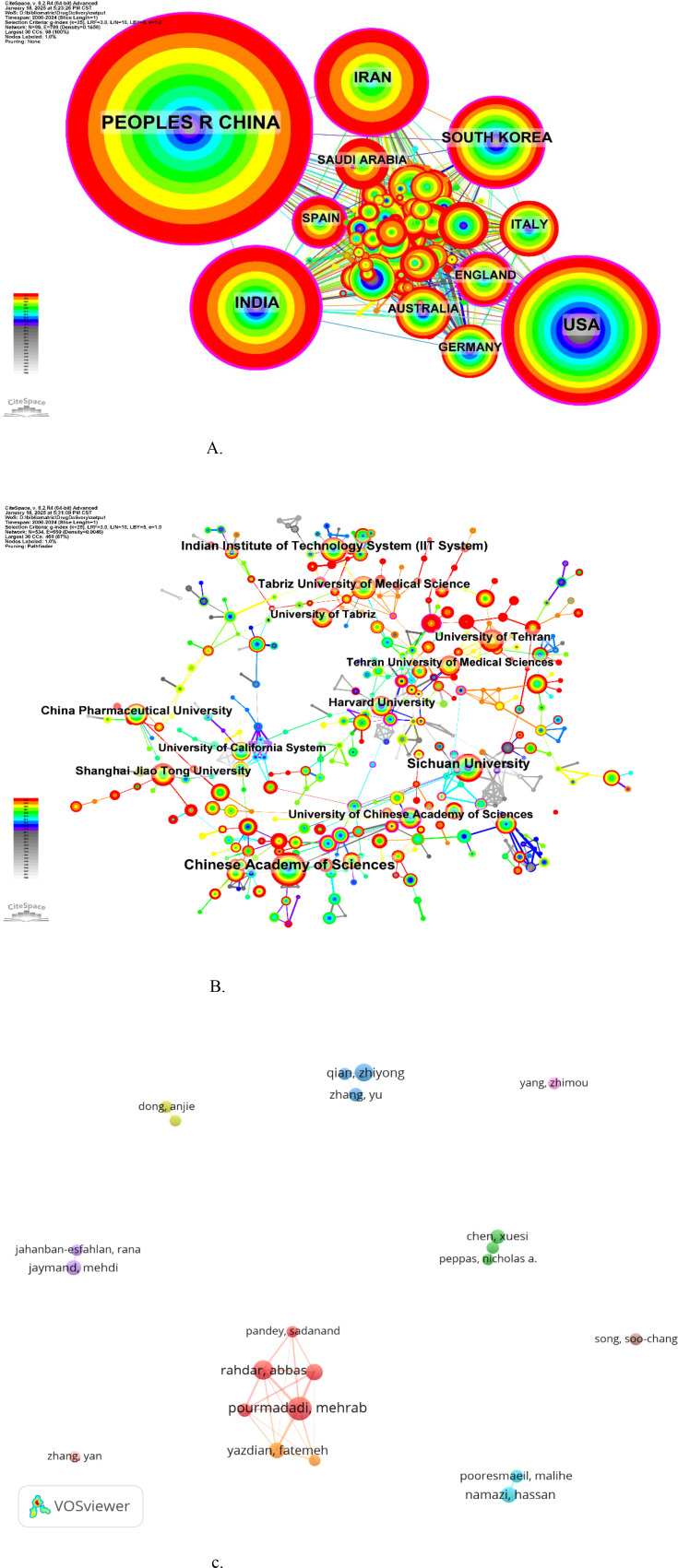
**(A)** The cooperation network of countries. **(B)** The cooperation network of institutions. **(c)** The cooperation network of authors.

**Figure 5** presents the scientific cooperation networks for countries, institutions, and authors, generated using CiteSpace and VOSviewer. The national collaboration network (**Figure 5A**) comprised 98 nodes and 788 links, with node size reflecting the volume of publications, ranging from China (largest) to the United States, India, Iran, and South Korea. The institutional collaboration network (**Figure 5B**) consists of 534 nodes and 660 links, with the Chinese Academy of Sciences, Sichuan University, the Indian Institutes of Technology System (IIT System), and Tabriz University of Medical Sciences representing prominent nodes. The author collaboration network is shown in **Figure 5C**. Pourmadadi Mehrab, Rahdar Abbas, Qian Zhiyong, and Abdouss Majid exhibited the highest publication counts in this field. The dense interconnections among these authors signify substantial collaborative activity (**Supplementary Table S1** in the **Supplementary Materials**). Notably, a clustering effect was observed among the nodes representing Pourmadadi Mehrab, Rahdar Abbas, and Pandey Sadanand, indicating a close collaborative group. Similarly, Qian Zhiyong, Zhang Yu, and their co-authors formed another distinct cluster.

### 3.2 Variation of the most active topics

#### 3.2.1 Subject category burst

**Figure 6:**
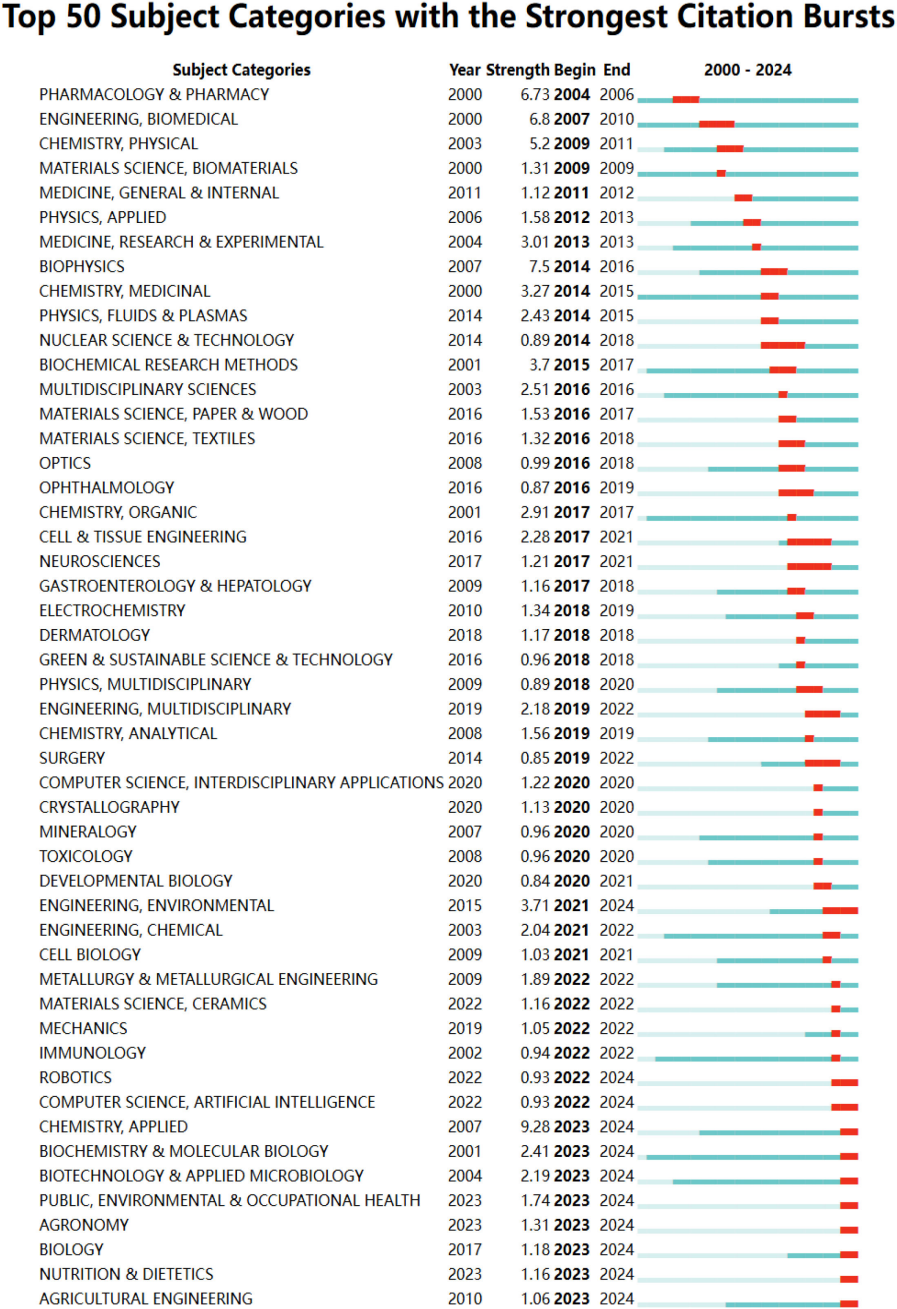
Top50 subject categories with the strongest citation bursts.

Of the 98 relevant categories, 94 experienced citation bursts between 2000 and 2024. Blue lines indicate the time interval, while red portions highlight the burst timeframe. **Figure 6** presents the top 50 categories with the highest burst intensity over time. The “Engineering, Biomedical” category exhibited a burst between 2007 and 2010, with a peak intensity of 6.8. Notably, the subject category bursts have diversified over time, as seen with “Chemistry, Physical” (2009-2011), “Physics, Applied” (2012-2013), “Nuclear Science & Technology” (2014-2018), “Physics, Multidisciplinary” (2018-2020), and “Computer Science, Artificial Intelligence” (2022-2024). This shift in emergent categories reflects the multidisciplinary nature of the field. In addition, 20 emergent categories originated in 2020 or later (**Supplementary Table S2** in the **Supplementary Materials**). The top three, based on recent burst activity, are “Chemistry, Applied” (2023-2024), “Engineering, Environmental” (2021-2024), and “Biochemistry & Molecular Biology” (2023-2024).

#### 3.2.2 Keyword burst

**Figure 7:**
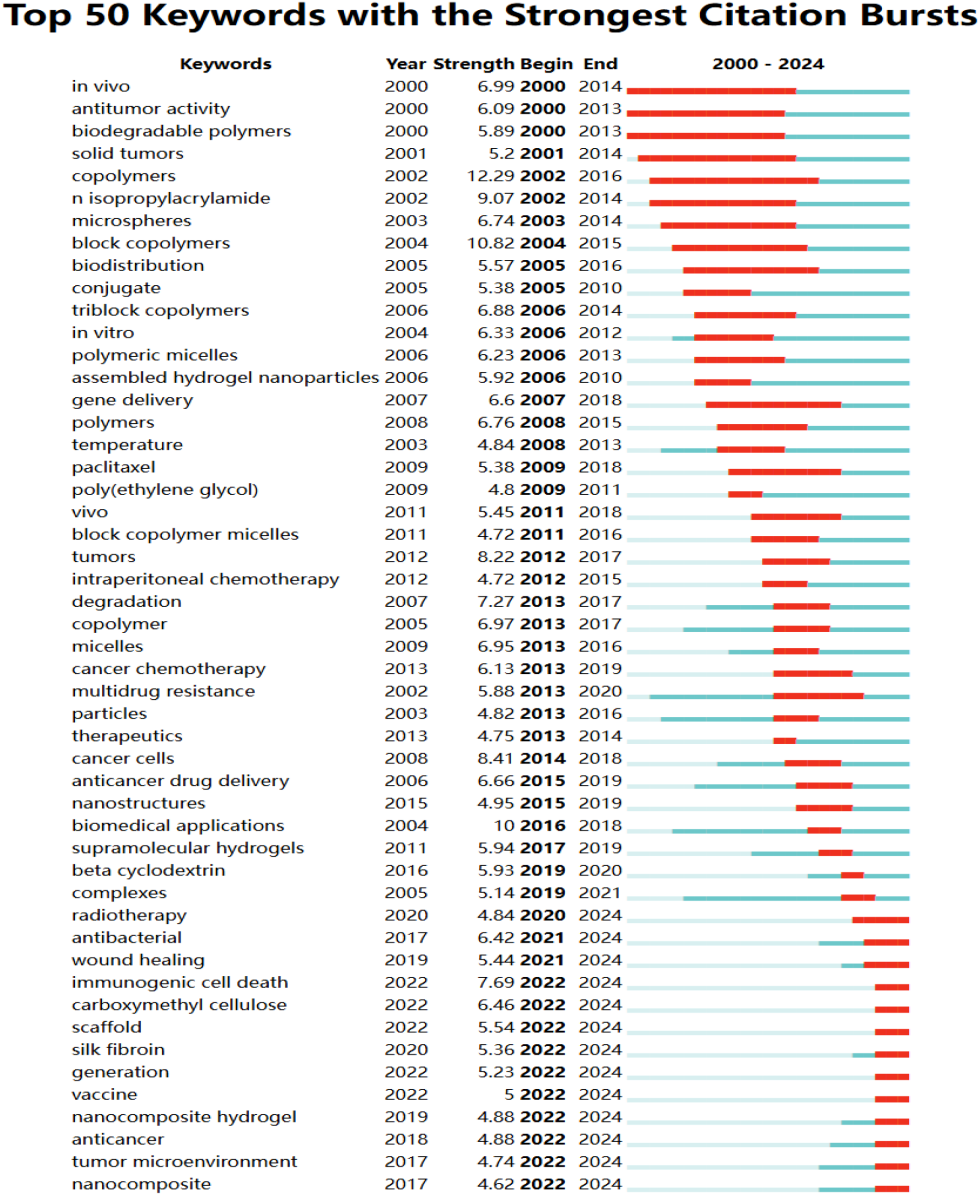
Top50 keywords with the strongest citation bursts.

A more detailed analysis of active hydrogel content within oncology drug delivery was conducted by examining keyword burst patterns from 2000 to 2024. Of the 759 keywords that exhibited bursts at various times, the top 50 with the highest burst intensity are presented in **Figure 7**. “Copolymers” showed the highest burst intensity (12.29) between 2002 and 2016, followed by “block copolymers” (10.82) between 2004 and 2015, and “n-isopropylacrylamide” (9.07) between 2002 and 2014. Notably, 20 keywords remained active in 2024, suggesting potential future research hotspots. Examples include “immunotherapy” (11.07, 2023-2024), “immunogenic cell death” (7.69, 2022-2024), “carboxymethyl cellulose” (6.46, 2022-2024), and “antibacterial” (6.46, 2022-2024). The burst intensity for “antibacterial” specifically between 2021 and 2024 was 6.42 (**Supplementary Table S2**, **Supplementary Materials**).

#### 3.2.3 Reference burst analysis

A total of 1,534 references were identified as having burst citations. The 30 most frequently cited references between 2000 and 2024 are shown in **Table 3**. Li JY (2016) (17) exhibited the highest citation burst rate, spanning from 2019 to 2021. The article concluded that hydrogel drug delivery systems provide beneficial therapeutic effects and have been used in clinical settings. Mura S (2013) (27) experienced a citation burst from 2015 to 2018, noting the recent surge in interest in advanced nanoscale drug delivery systems, particularly within nanomedicine. Norouzi M (2016) (20) had the third highest citation burst rate, attracting considerable attention upon publication and continuing for four years, from 2017 to 2021.

**Table 3:** The references with citation bursts at different period.

References	Year	Strength	Begin	End	2004 - 2024
Hoare TR, 2008, POLYMER, V49, P1993, DOI 10.1016/j.polymer.2008.01.027	2008	15.83	2010	2013	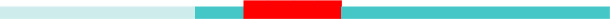
Bhattarai N, 2010, ADV DRUG DELIVER REV, V62, P83, DOI 10.1016/j.addr.2009.07.019	2010	12.11	2010	2015	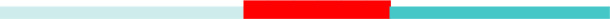
Hoffman AS, 2012, ADV DRUG DELIVER REV, V64, P18, DOI 10.1016/j.addr.2012.09.010	2012	18.47	2012	2017	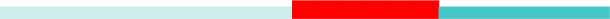
Kabanov AV, 2009, ANGEW CHEM INT EDIT, V48, P5418, DOI 10.1002/anie.200900441	2009	11.32	2012	2014	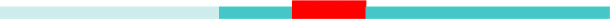
Wolinsky JB, 2012, J CONTROL RELEASE, V159, P14, DOI 10.1016/j.jconrel.2011.11.031	2012	16.58	2013	2017	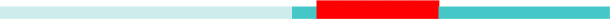
Mura S, 2013, NAT MATER, V12, P991, DOI 10.1038/nmat3776	2013	23.77	2015	2018	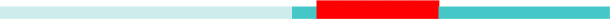
Qiu Y, 2012, ADV DRUG DELIVER REV, V64, P49, DOI 10.1016/j.addr.2012.09.024	2012	11.04	2015	2017	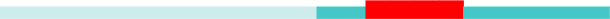
Ahmed EM, 2015, J ADV RES, V6, P105, DOI 10.1016/j.jare.2013.07.006	2015	19.36	2016	2020	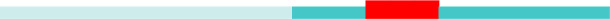
Fakhari A, 2015, J CONTROL RELEASE, V220, P465, DOI 10.1016/j.jconrel.2015.11.014	2015	14.6	2016	2020	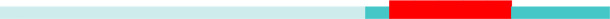
Norouzi M, 2016, DRUG DISCOV TODAY, V21, P1835, DOI 10.1016/j.drudis.2016.07.006	2016	22.33	2017	2021	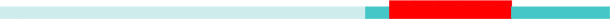
Du XW, 2015, CHEM REV, V115, P13165, DOI 10.1021/acs.chemrev.5b00299	2015	16.83	2017	2019	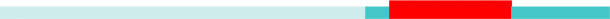
Conde J, 2016, NAT MATER, V15, P1128, DOI 10.1038/nmat4707	2016	14.08	2017	2021	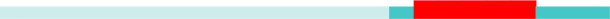
Merino S, 2015, ACS NANO, V9, P4686, DOI 10.1021/acsnano.5b01433	2015	12.98	2017	2020	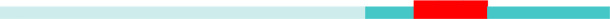
Qu Y, 2015, NPG ASIA MATER, V7, P0, DOI 10.1038/am.2015.83	2015	12.55	2017	2020	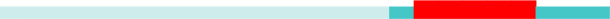
Bastiancich C, 2016, J CONTROL RELEASE, V243, P29, DOI 10.1016/j.jconrel.2016.09.034	2016	12.43	2017	2021	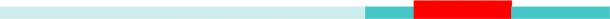
Blanco E, 2015, NAT BIOTECHNOL, V33, P941, DOI 10.1038/nbt.3330	2015	11.68	2017	2020	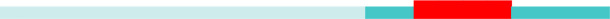
Shi JJ, 2017, NAT REV CANCER, V17, P20, DOI 10.1038/nrc.2016.108	2017	12.4	2018	2022	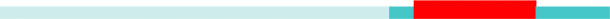
Ma HC, 2015, ACS APPL MATER INTER, V7, P27040, DOI 10.1021/acsami.5b09112	2015	7.88	2018	2019	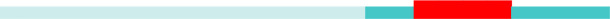
Li J, 2016, NAT REV MATER, V1, P0, DOI 10.1038/natrevmats.2016.71	2016	50.36	2019	2021	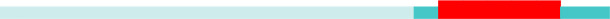
Qu J, 2017, ACTA BIOMATER, V58, P168, DOI 10.1016/j.actbio.2017.06.001	2017	15.28	2019	2022	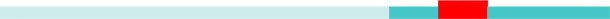
Wilhelm S, 2016, NAT REV MATER, V1, P0, DOI 10.1038/natrevmats.2016.14	2016	13.32	2019	2021	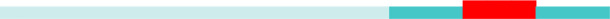
Senapati S, 2018, SIGNAL TRANSDUCT TAR, V3, P0, DOI 10.1038/s41392-017-0004-3	2018	11.22	2019	2024	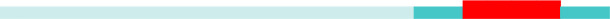
Chen Q, 2019, NAT NANOTECHNOL, V14, P89, DOI 10.1038/s41565-018-0319-4	2019	11.82	2020	2022	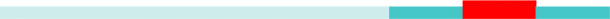
Dimatteo R, 2018, ADV DRUG DELIVER REV, V127, P167, DOI 10.1016/j.addr.2018.03.007	2018	10.97	2020	2024	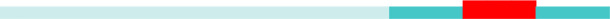
Sung H, 2021, CA-CANCER J CLIN, V71, P209, DOI 10.3322/caac.21660	2021	25.13	2022	2024	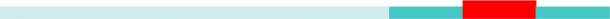
Sun ZY, 2020, MOL PHARMACEUT, V17, P373, DOI 10.1021/acs.molpharmaceut.9b01020	2020	21.31	2022	2024	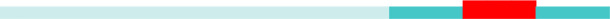
Mitchell MJ, 2021, NAT REV DRUG DISCOV, V20, P101, DOI 10.1038/s41573-020-0090-8	2021	15.27	2022	2024	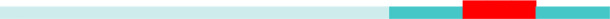
Cao H, 2021, SIGNAL TRANSDUCT TAR, V6, P0, DOI 10.1038/s41392-021-00830-x	2021	14	2023	2024	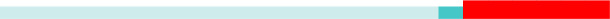
Correa S, 2021, CHEM REV, V121, P11385, DOI 10.1021/acs.chemrev.0c01177	2021	13.03	2023	2024	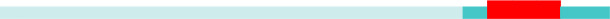
Zavareh HS, 2020, INT J BIOL MACROMOL, V165, P1422, DOI 10.1016/j.ijbiomac.2020.09.166	2020	11.27	2023	2024	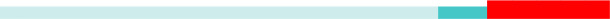

From 2004 onwards, 187 references exhibited citation bursts, with the top 20 strongest bursts detailed in **Table 4**. Of these, six were review articles and 14 were original research articles. Notably, all these publications experienced citation bursts either in the year of publication or the following year. The review articles provide valuable guidance for research on hydrogels in oncology drug delivery, while the research articles serve as excellent references for applications of hydrogels in this field. This observation highlights the importance of researchers in the field of hydrogels for oncology drug delivery paying close attention to this rapidly evolving body of literature.

**Table 4:** The references with citation bursts from beginning to 2024.

begin	end	Strength	Year	Type	Title
2022	2024	21.31	2020	Review	Hydrogel-Based Controlled Drug Delivery for Cancer Treatment: A Review
2022	2024	15.27	2021	Review	Engineering precision nanoparticles for drug delivery
2023	2024	14	2021	Review	Current hydrogel advances in physicochemical and biological response-driven biomedical application diversity
2023	2024	13.03	2021	Review	Translational Applications of Hydrogels
2023	2024	11.27	2020	Article	Chitosan/carbon quantumdot/aptamer complex as a potential anticancer drug delivery system towards the release of 5-fluorouracil
2019	2024	11.22	2018	Review	Controlled drug delivery vehicles for cancer treatment and their performance
2020	2024	10.97	2018	Review	*In situ* forming injectable hydrogels for drug delivery and wound repair
2022	2024	10.25	2021	Article	Injectable thermosensitive hydrogel-based drug delivery system for local cancer therapy
2023	2024	10.1	2022	Article	Novel Carboxymethyl Cellulose-Based Hydrogel with Core-Shell Fe3O4@SiO2 Nanoparticles for Quercetin Delivery
2022	2024	9.69	2020	Review	Hydrogels in the clinic
2020	2024	9.4	2018	Review	Nano based drug delivery systems: recent developments and future prospects
2023	2024	8.96	2021	Article	Ameliorating quercetin constraints in cancer therapy with pH-responsive agarose-polyvinylpyrrolidone -hydroxyapatite nanocomposite encapsulated in double nanoemulsion
2023	2024	8.96	2022	Article	Preparation of a pH-responsive chitosan-montmorillonite-nitrogen-doped carbon quantum dots nanocarrier for attenuating doxorubicin limitations in cancer therapy
2023	2024	8.87	2020	Review	Fundamental Concepts of Hydrogels: Synthesis, Properties, and Their Applications
2023	2024	8.54	2021	Article	Synthesis and characterization of chitosan/polyvinylpyrrolidone coated nanoporous γ-Alumina as a pH-sensitive carrier for controlled release of quercetin
2020	2024	8.35	2019	Review	Photothermal therapy and photoacoustic imaging via nanotheranostics in fighting cancer
2022	2024	8.3	2021	Review	Preparation of pH-sensitive chitosan/polyvinylpyrrolidone/α-Fe2O3 nanocomposite for drug delivery application: Emphasis on ameliorating restrictions
2022	2024	8.3	2021	Review	Functional Hydrogels as Wound Dressing to Enhance Wound Healing
2022	2024	8.29	2020	Review	Hydrogel-By-Design: Smart Injectable Hydrogels for Cancer Immunotherapy
2021	2024	8.24	2019	Review	Injectable Hydrogels for Cancer Therapy over the Last Decade

### 3.3 Emerging trends and new developments

#### 3.3.1 The temporal variation of keyword clusters

**Figure 8:**
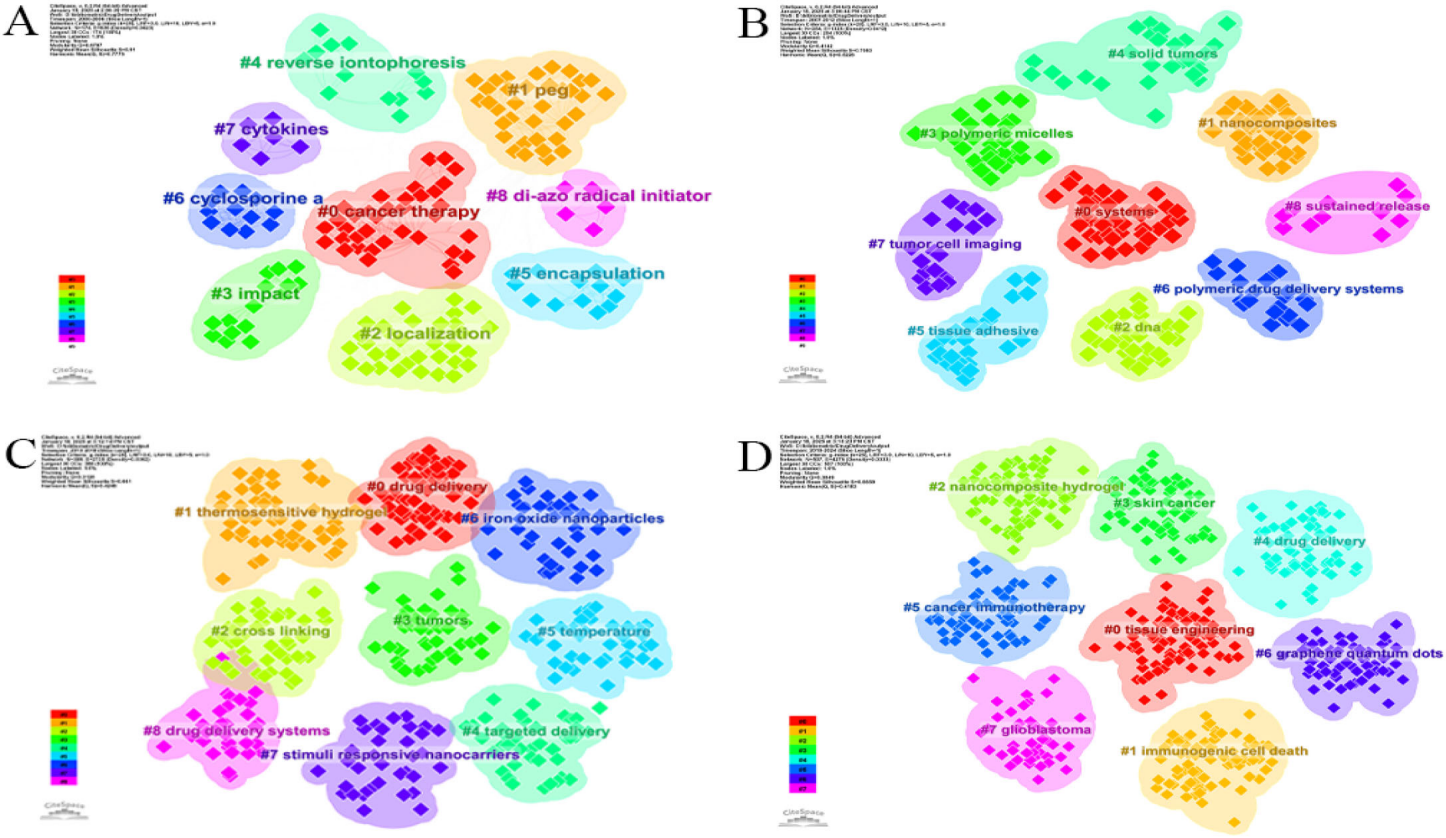
Knowledge map of keyword clustering. **(A)** 2000-2006, **(B)** 2007-2012, **(C)** 2013-2018, **(D)** 2019-2024.

There exists a close relationship between different keywords. These keywords can generate different clusters, and the identification of these clusters can indicate various subfields of hydrogel in cancer drug delivery research. The past 24 years were divided into four six-year phases, with keyword clustering snapshots for each phase shown in **Figure 8**. The first clustering (2000-2006), based on 54 papers, yielded 9 clusters, including #0 cancer therapy, #1 polyethylene glycol (PEG), and #2 localization (**Figure 8A**). The second clustering (2007-2012), analyzing 298 papers, produced 11 clusters, including #0 systems, #1 nanocomposites, and #2 DNA (**Figure 8B**). The third clustering (2013-2018), encompassing 1105 papers, generated 9 clusters, such as #0 drug delivery, #1 thermosensitive hydrogel, #2cross linking, and 9 other clusters (**Figure 8C**). The fourth clustering (2019-2024), including 2651 papers, resulted in 8 clusters, including #0 tissue engineering, #1 immunogenic cell death, and #2 nanocomposite hydrogel (**Figure 8D**). While classical areas like cancer therapy and drug delivery remain active research areas, emerging clusters such as #0 tissue engineering, #1 immunogenic cell death, #2 nanocomposite hydrogel, #3 skin cancer, #5 cancer immunotherapy, #6 graphene quantum dots, and #7 glioblastoma have gained increasing attention compared to the previous 15 years.

Analysis of emerging literature clusters revealed a focus on key areas within hydrogel research for oncology drug delivery. Cluster 0#, “tissue engineering,” comprises 77 articles. Cluster 1#, “immunogenic cell death,” and Cluster 5#, “cancer immunotherapy,” contain 76 and 58 articles, respectively, reflecting research into various cell death mechanisms and the role of hydrogels in tumor immunotherapy. Cluster 2#, “nanocomposite hydrogel,” and Cluster 6#, “graphene quantum dots,” with 71 and 56 articles, respectively, explore different nanomaterials for tumor drug delivery. Cluster 3#, “skin cancer,” and Cluster 7#, “glioblastoma,” with 61 and 47 articles, respectively, examine the application of hydrogels in treating specific tumor types. **Supplementary Table S3** (**Supplementary Materials**) provides detailed data for the fourth clustering period (2018-2024), including “representative keywords within the clusters” that identify core research areas within the most recent phase of hydrogel research for oncology drug delivery.”

#### 3.3.2 The keyword alluvial flow visualization

**Figure 9:**
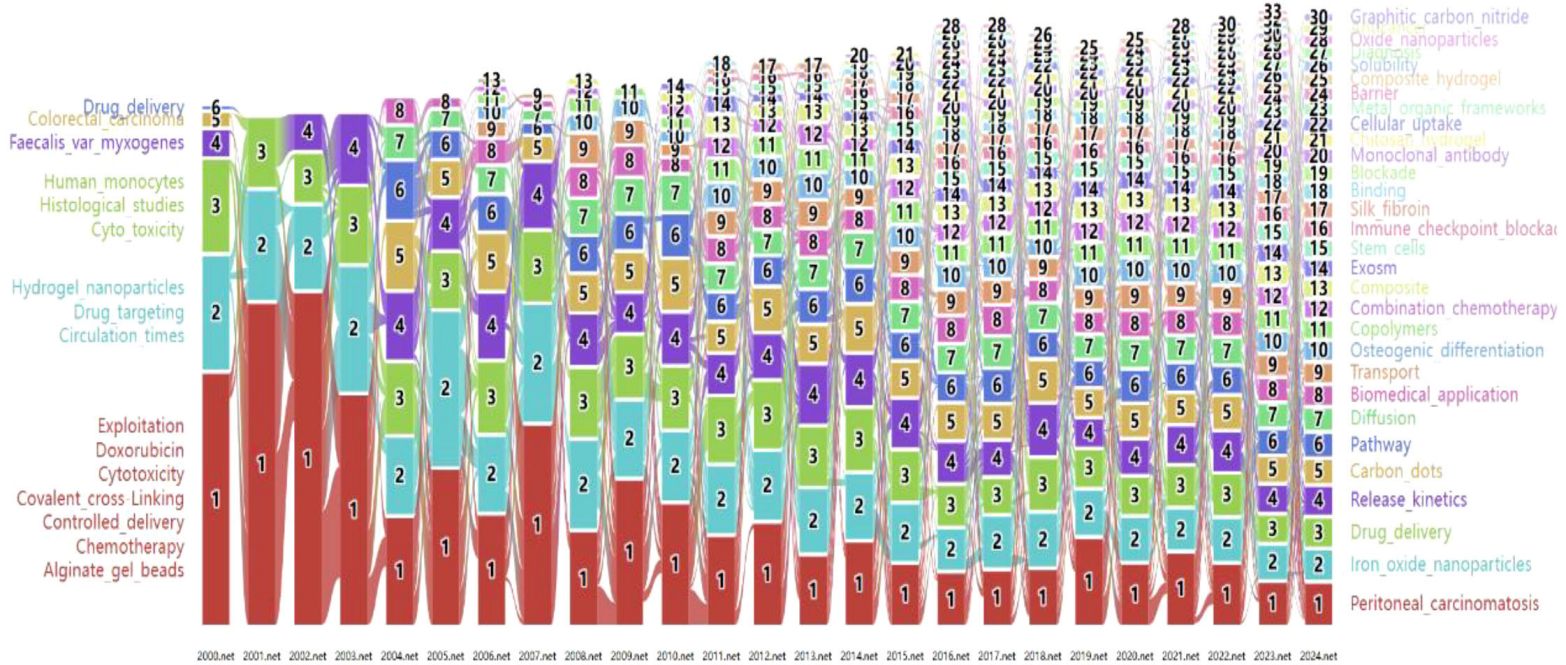
The keywords alluvial map 2000–2024. X axis: Time slice. Y axis: Counting of modules.

As shown in **Figure 9**, linked keywords form specific research modules, and keywords form new modules over time. Over the past 25 years, some high-traffic keywords have persisted, some have increased in importance and popularity, while others have disappeared. **Supplementary Table S4** (**Supplementary Materials**) lists the top five highest-traffic module keywords for each year. Notably, the keywords within Module 1 in 2024 diverged or converged at this research turning point, forming the largest research tributary (highlighted in red). This suggests that Module 1 represents the most enduring research module. **Figure 2** maps all keywords for the top six modules in 2024. Module 1, designated “peritoneal_carcinomatosis”, encompasses 21 keywords, such as tumor growth, therapeutic efficacy, and esophageal cancer (**Figure 2A**). Module 2, named “iron_oxide_nanoparticles”, contains 13 keywords, such as DNA, ferroptosis, efficiency, and antibody (**Figure 2B**). Module 3, “drug_delivery”, comprises 16 keywords such as nanoparticles, release, *in vitro*, and pH (**Figure 2C**). Module 4, “release_kinetics”, contains 8 keywords, such as cytotoxicity, stability, chitosan nanoparticles, and polyethylene glycol (**Figure 2D**). Module 5, “carbon_dots”, includes 13 keywords, such as floating hydrogel, classification, and cyclodextrin (**Figure 2E**). Finally, Module 6, “pathway”, encompasses 13 keywords, including extraction, metastasis, derivatives, and proliferation (**Figure 2F**). These modules likely represent emerging trends in hydrogels for oncology drug delivery over the next five years and potentially beyond.

#### 3.3.3 The timeline visualization of references

**Figure 10:**
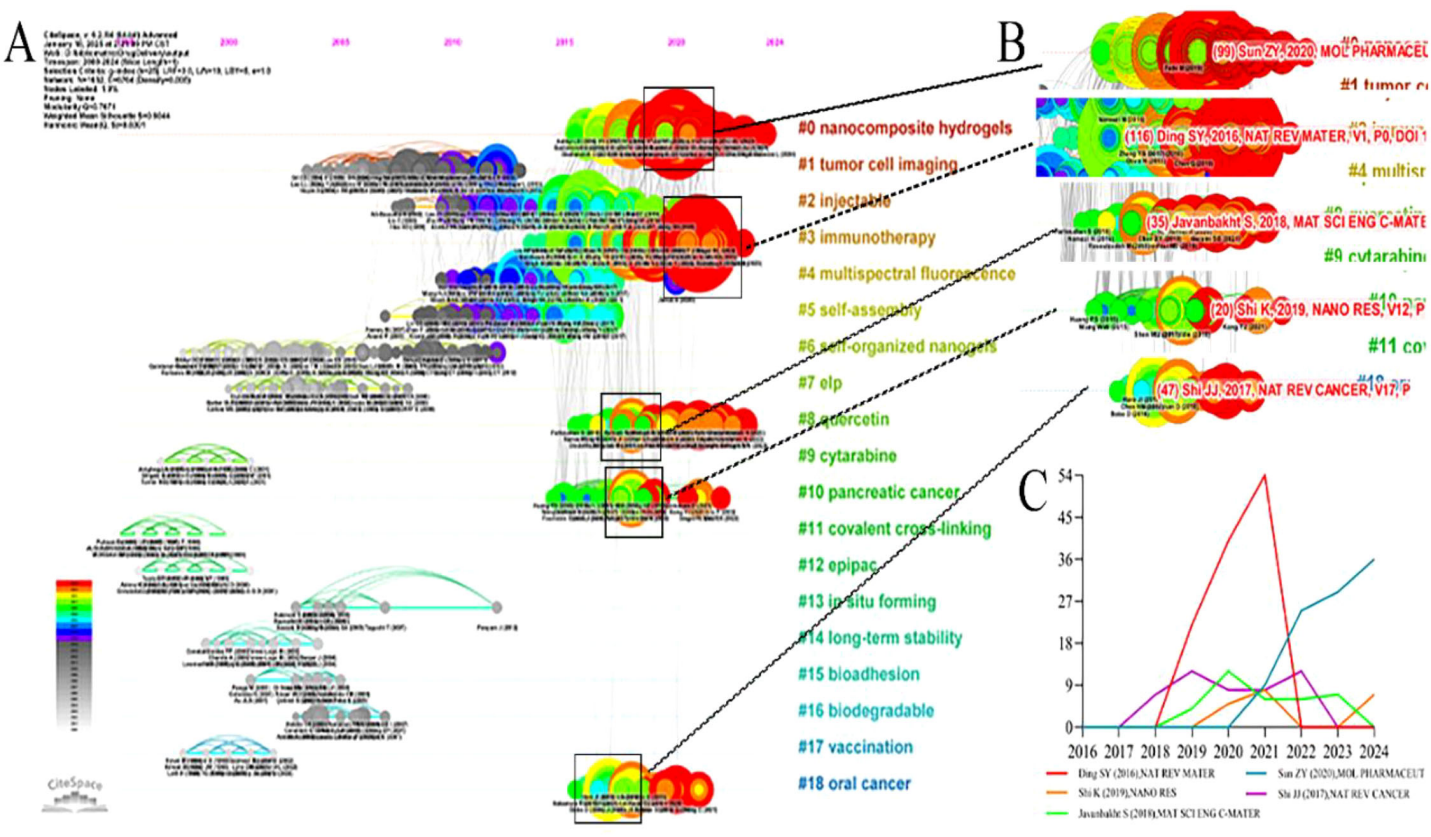
The reference clusters map. **(A)** the citation timeline visualization, **(B)** The burst citation in #0, #3, #8, #10, and #18, **(C)** citation frequency distribution of the burst citation, X-axis: Year, Y-axis: Cited frequency.

A timeline visualization, based on citation time spans, identifies emerging, classic, and outdated research topics. The timeline plot of hydrogel studies in oncology drug delivery comprised 19 clusters, arranged in descending order of size (**Figure 10A**). Clusters #1 (tumor cell imaging), #2 (injectable), #4 (multispectral fluorescence), and #5 (self-assembly) represent classic topics. While not necessarily the most recent, these topics were intrinsically linked to other clusters. Clusters #6 (self-organized nanogels), #7 (elp), #9 (cytarabine), #11 (covalent cross-linking), #12 (cytarabine), #13 (*in situ* forming), #14 (long-term stability), #15 (bioadhesion), #16 (biodegradable), and #17 (vaccination) are considered relatively outdated, exhibiting few connections to other clusters and limited subsequent development. Clusters #0 (nanocomposite hydrogels), #3 (immunotherapy), #8 (quercetin), #10 (pancreatic cancer), and #18 (oral cancer) were emerging topics, demonstrating continued activity on the timeline since their emergence. This suggested their potential to become future research hotspots. **Supplementary Table S5** (**Supplementary Materials**) provides further details on these emerging clusters. In addition, some classical papers (large nodes with red circles) played a very important role in advancing the subfields (**Figure 10B**).

Cirillo G’s 2019 publication, “Injectable Hydrogels for Cancer Therapy over the Last Decade”, was attributed to group 0 and exhibited a co-citation frequency of 99 (28). This article highlighted research efforts over the past decade focused on injectable hydrogels for cancer therapy. Many researchers are developing sophisticated injectable drug delivery systems suitable for combination chemotherapy, radiation therapy, and thermal and photothermal ablation, aiming to overcome limitations of current conventional treatments.

The study “Designing hydrogels for controlled drug delivery” (15), published by Li JY, belonged to group 3 and exhibited a co-occurrence frequency of 116. This research demonstrated the ability of hydrogels to control drug release both spatially and temporally, encompassing various drug types such as small molecule drugs, large molecule drugs, and cells. Hydrogels, possessing controlled degradability and the capacity to protect labile drugs from degradation, can be used as a platform on which interactions with encapsulated drugs occur to achieve controlled drug release. They also presented different mechanisms for the design of hydrogel drug delivery systems, especially the physical and chemical properties and the interaction of the hydrogel with the drug at the network, reticular and molecular scales. In addition, they discussed the interplay and integration of these mechanisms for precise spatiotemporal control of drug release.

Javanbakht S authored a publication titled “Doxorubicin loaded carboxymethyl cellulose/graphene quantum dot nanocomposite hydrogel films as a potential anticancer drug delivery system”, which belonged to cluster 8 and exhibited a co-occurrence frequency of 35 (29). In this study, they designed a novel hydrogel nanocomposite film with anticancer properties by incorporating graphene quantum dot (GQD) nanoparticles in carboxymethyl cellulose (CMC) hydrogel with doxorubicin (DOX) as a drug model. The prepared nanocomposite hydrogel films showed improved *in vitro* solubility, degradation, water vapor permeability and pH-sensitive drug delivery properties. The nanocomposite hydrogel films could be used as anticancer films and drug delivery systems.

The article “Functional hydrogels as wound dressing to enhance wound healing” by Liang YP et al. (30) was attributed to group 3. Hydrogels possess favorable biochemical and mechanical properties, offering significant advantages in wound dressing applications. This paper mainly introduced the antibacterial, anti-inflammatory, and antioxidant properties of hydrogel dressings, along with their material transport and self-healing capabilities. It also summarized the application of hydrogel wound dressing in the treatment of different types of wounds, demonstrating their effectiveness in promoting wound healing.

Shi K et al. (31) reported on the sustained co-delivery of gemcitabine and cisplatin using a biodegradable, thermo-sensitive hydrogel for synergistic combination therapy of pancreatic cancer. They developed a biodegradable, thermosensitive copolymer hydrogel for the combined delivery of gemcitabine (GEM) and cisplatin (DDP) in pancreatic cancer treatment. This hydrogel exists as a free-flowing liquid at room temperature, transitioning to a semi-solid state at physiological temperatures upon injection. *In vitro* and *in vivo* drug release studies demonstrated sustained drug release from this system. The hydrogel system exhibited a stronger synergistic therapeutic effect against pancreatic cancer, both *in vitro* and *in vivo*, compared to hydrogels loaded with single drugs.

Shi et al.’s 2017 publication, “Cancer nanomedicine: progress, challenges and opportunities” (32), was attributed to group 18. The limitations of traditional cancer therapies have spurred in-depth research into nanotechnology for more effective and safer cancer treatment, giving rise to the field of cancer nanomedicine. This study primarily reviewed the development of cancer nanomedicine, concluding that a deeper understanding of tumor biology and nanobiology is crucial for developing improved cancer nanotherapies. Analysis of the citation distribution of these five articles over recent years (**Figure 10C**) suggested that these publications were likely to continue to be cited in the future.

## 4 Discussion

### 4.1 General information

Cancer is a complex and life-threatening disease with numerous, often imperfect, treatment options. Precisely delivering therapeutic drugs to tumor tissue for release can significantly improve cancer therapy efficacy. Drug delivery systems enhance drug pharmacokinetics and tumor site accumulation, playing a crucial role in cancer therapy (33). In recent years, hydrogels have been used as drug delivery systems in tumor therapy and have gained widespread attention due to their unique physical and chemical properties (34, 35).

This study analyzed hydrogels for drug delivery in cancer using bibliometric methods, employing CiteSpace, HisCite, the Alluvial Generator, and R software. The 4108 retrieved articles related to hydrogels in oncology drug delivery were published in 664 journals across 98 scientific categories. From 2014 to 2024, the number of annual publications related to hydrogels in cancer drug delivery consistently increased, exceeding 100 annually (**Figure 1A**). 2024 saw the highest number of publications, with 613 articles, clearly indicating growing interest in hydrogels as drug delivery systems in tumor therapy. The top three publishing journals were the *International Journal of Biological Macromolecules*, the *Journal of Drug Delivery Science and Technology*, and the *Journal of Controlled Release* (**Figure 3**). Researchers should therefore pay close attention to these journals. Co-occurrence frequency analysis identified highly influential countries, institutions, and active authors, providing valuable insights into the field of hydrogels for drug delivery in cancer. Among national collaborations, China, the United States, India, Iran, and South Korea frequently occupied central positions within international cooperation networks (**Figure 5A**), with China exhibiting the highest co-occurrence frequency. Analysis of institutional collaborations revealed primarily domestic collaborations, with limited direct and in-depth international exchange (**Figure 5B**). China also hosted the top two institutions, the Chinese Academy of Sciences and Sichuan University, exhibiting high co-occurrence frequency in this field (**Supplementary Table S1**). In addition, the analysis revealed a limited number of high-yield authors and a low degree of collaboration among them.

### 4.2 Research topics and trends

Burst detection of subject categories and keywords from 2000 to 2024 was performed to analyze the development trends and evolving characteristics of hydrogels in tumor drug delivery. Polypeptide self-assembled materials, a type of synthetic biomaterial, offer advantages such as simple synthesis, good biocompatibility, low immunogenicity, and low toxicity (36). Hydrogels, composed of hydrophilic polymers, possess a three-dimensional network structure that allows for significant water absorption and drug carrying capacity (37). The burst period for the biomedical discipline occurred between 2007 and 2010, indicating an early focus on biomedical applications (**Figure 6**). Over time, the research focus has shifted towards chemistry, engineering, environmental science, and biochemistry & molecular biology. Hydrogels can also be combined with metal-organic frameworks (MOFs) to create composite materials, forming stimulus-responsive local drug delivery systems with dynamic structural characteristics. This approach enables localized and sustained drug release *in vivo*, effectively inhibiting tumor growth (38). In contrast to conventional substrates, biochemically responsive hydrogels modulate the interplay between biological events, hydrogel properties, and cellular behaviors (39). Interdisciplinary research on hydrogels enhances our understanding of their application potential in tumor drug delivery and promotes further advancements in cancer treatment. Keyword burst analysis, based on frequently occurring keywords within specific time periods, revealed research hotspots and trends in hydrogels for tumor drug delivery. Current research keywords identified include immunotherapy, immunogenic cell death, carboxymethyl cellulose, and antibacterial, reflecting current research hotspots in the field.

#### 4.2.1 Immunotherapy

Conventional tumor therapy focuses on the tumor itself, but given the complexity of the tumor microenvironment, immunotherapy has emerged as a promising approach. Immunotherapy leverages the body’s immune system to attack tumors, wherein immune cells target and destroy tumor cells within the tumor microenvironment (40, 41). Tumor immunotherapy is a guideline-recommended treatment modality for gastrointestinal tumors, demonstrating good efficacy and fewer side effects (42). Hydrogels, capable of drug loading, controlled drug release, and delivery of immunomodulatory molecules, immune cells, and environmental regulators, can improve the overall response rate of immunotherapy. This has led to their emergence as multifunctional materials in biomedical applications (43). Yang Y et al. developed a shear-thinning injectable hydrogel that enhances local immunotherapy for gastric cancer by repolarizing tumor-associated macrophages (44). Their results demonstrated favorable tumor growth inhibition after a single injection. Similarly, Seo HS et al. developed a multifunctional ethylene glycol methacrylate-chitosan (MGC) hydrogel that effectively inhibited tumor recurrence and metastasis, enhanced immunotherapy efficacy, and minimized side effects (45). These findings indicate that hydrogel-based drug delivery systems combined with cancer immunotherapy could significantly improve local and systemic treatment outcomes, providing more precise and effective treatment strategies.

#### 4.2.2 Immunogenic cell death

Immunogenic cell death (ICD) is a form of programmed cancer cell death wherein dying cancer cells release immune-stimulating factors, prompting the immune system to recognize and eliminate tumors (46). ICD not only promotes the release of tumor-associated antigens but also activates the immune response of T cells through the maturation of antigen-presenting cells (47). The occurrence of ICD is characterized by changes in surface molecules, the release of inflammatory factors, and antigen activation, all of which contribute to enhancing the anti-tumor response of the immune system. Studies have shown that nanomaterials can enhance tumor immunogenicity by inducing ICD, thereby improving the efficacy of immunotherapy (48). Shen et al. designed a copper (Cu)-induced injectable hydrogel. This ion-induced self-assembled hydrogel containing NO can enhance immunotherapy by amplifying ICD and regulating cancer-associated fibroblasts (49). A pH-responsive polyamidoamine dendritic nanocarrier-incorporated hydrogel can serve as a drug delivery system to bind immunochemotherapy drugs, induce immunogenic cell death-related cytokines, and trigger CD8^+^ T cell-mediated immune responses to enhance immunogenic cell death and reverse immunosuppression, leading to significant tumor growth inhibition (50). An in-depth study of ICD will improve our understanding of the role of hydrogel drug delivery systems in the tumor immune microenvironment and facilitate the development of more effective immunotherapy strategies.

#### 4.2.3 Cellulose

Cellulose, a renewable polymer, and its derivatives are widely used in various medical applications, including tissue engineering, artificial blood vessels, and drug carriers, with many applications related to the treatment of cancer (51). Carboxymethyl cellulose (CMC), a readily available chemical product, possesses thickening and relative stability properties, making it an ideal hydrogel carrier (52). This hydrogel exhibits biocompatibility, biodegradability, and antibacterial properties. CMC hydrogel can deliver the anticancer drug 5-fluorouracil, and adjusting the electric field can influence the drug’s diffusion coefficient, enabling precise drug release (53). Combining CMC with other materials can further enhance hydrogel properties. Nano-composite hydrogel beads formed by combining CMC with starch and ZnO nanoparticles have been investigated for the controlled release of doxorubicin. This composite material can prolong the drug release and control it more accurately by adjusting the content of ZnO nanoparticles (54). Nanocomposite hydrogel beads composed of CMC, starch, and zinc oxide nanoparticles (ZnO-NPs) have also been studied for the controlled release of doxorubicin. Increasing the ZnO nanoparticle content can prolong drug release time, and this composite has also demonstrated toxicity to human colon cancer cells *in vitro* (54). CMC-based hydrogels have diverse applications in drug delivery systems and can achieve precise drug release control and enhanced biocompatibility through various combinations and modifications.

#### 4.2.4 Antimicrobial hydrogels

In recent years, with increased understanding of tumor biology, tumor-targeted drugs have seen rapid development in cancer treatment. However, viral reactivation can occur in patients undergoing targeted therapy (55), necessitating viral prophylaxis and anti-inflammatory measures post-treatment. The development and design of bioactive materials with both anti-tumor and anti-bacterial properties has gained attention due to the established link between bacteria and tumors. Antimicrobial hydrogels possess excellent antimicrobial properties, good biocompatibility, water absorption and retention, and high oxygen permeability, making them widely applicable in biomedicine, smart textiles, cosmetics, and medical dressings (56, 57). By modifying the properties of hydrogel, its antibacterial, vascularization, anti-inflammatory and antioxidant properties can be enhanced (58). In one study, a nanocomposite dual network (NDN) hydrogel with tumor-targeting effects was found to improve the therapeutic efficacy of radiation therapy and eliminate potentially pathogenic bacteria to prevent postoperative wound infections (59).

#### 4.2.5 Chitosan

Chitosan, the only naturally occurring alkaline polysaccharide, is derived from chitin through deacetylation and possesses immunomodulatory, anti-cancer, and lipid metabolism-regulating properties (60). Its favorable biocompatibility, biodegradability, and blood compatibility make chitosan hydrogels widely applicable in medicine (61). One study developed a chitosan-based thermosensitive hydrogel containing adriamycin liposomes for localized cancer therapy. This hydrogel rapidly forms a gel at body temperature, significantly enhancing anti-tumor activity while reducing systemic toxicity (62). A modified chitosan thermosensitive hydrogel, administered via intratumoral injection, achieves sustained paclitaxel release and significantly enhances anti-tumor activity (63). Composite hydrogels, formed by combining chitosan with other materials, exhibit excellent drug-release properties and biocompatibility. For example, oxyamino chitosan, as a co-delivery carrier for genes and drugs, effectively facilitates anti-cancer therapy (64). Combining and functionally modifying chitosan hydrogels with other materials to enhance their potential in drug delivery systems leads to more effective oncology drug therapy.

#### 4.2.6 Nanohydrogels

Nanoscale drug delivery systems enhance the retention, accumulation, and penetration of immunotherapeutic drugs at the tumor site, while also enabling controlled drug release. Incorporating micron- and nanoscale materials into hydrogels can improve their mechanical and functional properties, resulting in composite gels. Nanohydrogels can be classified as either conventional or environmentally responsive based on their phase transition mechanisms (65). Environmentally responsive nanohydrogels can respond to various external stimuli, enabling controlled drug release, enhanced penetration and retention of chemotherapeutic drugs, and improved therapeutic efficacy (65). Furthermore, combining nanohydrogels with gene therapy via intelligent drug delivery systems can further enhance tumor therapy effectiveness (66). Composite hydrogels are versatile and tunable, finding wide applications in tumor model reconstruction, tumor diagnosis, and tumor therapy.

#### 4.2.7 Injectable hydrogels

Injectable hydrogel systems have applications in the postoperative management of tumors (67). Implantable hydrogel scaffolds can be designed for drug release or to facilitate immune cell infiltration. For example, an injectable, thermosensitive hydrogel based on black phosphorus nanosheets and adriamycin has been developed for photothermal chemotherapy of bone tumors, which can continuously release adriamycin to improve the anti-tumor efficacy and reduce systemic toxicity (68). Injectable hydrogels, prepared using appropriate physical and chemical crosslinking methods, possess both injectability and self-healing capabilities, achieving a balance between ease of preparation and material functionality. At present, clinically used hydrogel products are primarily administered via oral mucosa, oral, vaginal, transdermal, and ocular routes (69).

### 4.3 Strength and limitations

Unlike traditional review articles, this study employed bibliometric analysis to comprehensively examine hydrogel drug delivery systems in oncology therapy, considering institutional collaborations, subject categories, keywords, and highly cited literature. Compared to previous reviews, this approach identified and presented the most valuable articles and keywords using visual charts and graphs, providing a clear overview of the trajectory of hydrogel drug delivery systems in oncology therapy. Some high-quality review articles have a high citation rate, and their citation can reflect the influence and importance of related research in this field to some extent. However, this study has limitations that warrant further investigation. First, the WoSCC database was the sole data source. While the WoSCC database is continuously updated and provides authoritative, relevant, and comprehensive information, relying on a single database may introduce bias. Second, the use of multiple analysis software packages, each with its own filtering algorithms, may have resulted in the omission of some information, potentially influencing the results. Furthermore, some high-quality articles were published late, resulting in their low citation counts, and these need to be focused on in future research.

### 4.4 Conclusion

Overall, hydrogels have gained widespread use as emerging drug carriers for oncology drug delivery. This study characterized the distribution of publications, identified subject categories, keywords, and reference bursts, and highlighted developing trends and frontiers in hydrogels for cancer drug delivery. Over the past two decades, publications on hydrogels for cancer drug delivery have increased significantly, particularly since 2016, with annual publication numbers reaching 200. China and the Chinese Academy of Sciences ranked first in co-occurrence frequency. Emerging research directions include tumor immunotherapy, immunogenic cell death, carboxymethyl cellulose hydrogels, antimicrobial hydrogels, chitosan hydrogels, nanohydrogels, and hydrogels with varied delivery modes. Overall, this study provides researchers with potential guidance for future applications of hydrogels in the medical field.

## Data Availability

The original contributions presented in the study are included in the article/**Supplementary Material**. Further inquiries can be directed to the corresponding authors.
