# Supplementary material for: Hydrogels in cancer treatment: mapping the future of precision drug delivery
Source: Front Immunol. 2025 Jul 8;16:1607240. doi: 10.3389/fimmu.2025.1607240 (PMC12279752; doi:10.3389/fimmu.2025.1607240)
Supplement: Supplementary file 1 [file Table1.docx]

**Supplementary materials**

Table S1. The top 10 countries, institutions and authors for frequency of co-occurrence.

| **CoF** | **Year** | **Country** |  | **CoF** | **Year** | **Institution** |  | **CoF** | **Citation** | **Author** |
| --- | --- | --- | --- | --- | --- | --- | --- | --- | --- | --- |
| 1664 | 2004 | CHINA |  | 169 | 2009 | Chinese Academy of Sciences |  | 56 | 1190 | pourmadadi, mehrab |
| 715 | 2000 | USA |  | 117 | 2009 | Sichuan University |  | 39 | 572 | rahdar, abbas |
| 482 | 2000 | INDIA |  | 84 | 2010 | Indian Institute of Technology System (IIT System) |  | 32 | 1883 | qian, zhiyong |
| 373 | 2009 | IRAN |  | 77 | 2016 | Tabriz University of Medical Science |  | 28 | 370 | abdouss, majid |
| 291 | 2000 | SOUTH KOREA |  | 71 | 2013 | China Pharmaceutical University |  | 26 | 1365 | namazi, hassan |
| 133 | 2003 | ITALY |  | 70 | 2011 | Shanghai Jiao Tong University |  | 25 | 861 | yazdian, fatemeh |
| 114 | 2005 | ENGLAND |  | 67 | 2003 | Harvard University |  | 23 | 609 | jaymand, mehdi |
| 99 | 2007 | GERMANY |  | 62 | 2020 | University of Tehran |  | 22 | 1383 | chen, xuesi |
| 96 | 2006 | AUSTRALIA |  | 59 | 2013 | University of Chinese Academy of Sciences |  | 20 | 403 | diez-pascual, ana m. |
| 91 | 2011 | SAUDI ARABIA |  | 52 | 2003 | University of California System |  | 19 | 883 | zhang, yu |

CoF: Co-occurrence frequency. Year:Year of first collaboration appearance

Table S2. The top 20 subject categories and keywords burst with a burst period from beginning to 2024.

| **Subject category bursts** | | | | | **Keywords bursts** | | | | |
| --- | --- | --- | --- | --- | --- | --- | --- | --- | --- |
| Begin | End | Strength | Year | Entity | Begin | End | Strength | Year | Entity |
| 2023 | 2024 | 9.28 | 2007 | CHEMISTRY, APPLIED | 2023 | 2024 | 11.07 | 2018 | immunotherapy |
| 2021 | 2024 | 3.71 | 2015 | ENGINEERING, ENVIRONMENTAL | 2022 | 2024 | 7.69 | 2022 | immunogenic cell death |
| 2023 | 2024 | 2.41 | 2001 | BIOCHEMISTRY & MOLECULAR BIOLOGY | 2022 | 2024 | 6.46 | 2022 | carboxymethyl cellulose |
| 2023 | 2024 | 2.19 | 2004 | BIOTECHNOLOGY & APPLIED MICROBIOLOGY | 2021 | 2024 | 6.42 | 2017 | antibacterial |
| 2023 | 2024 | 1.74 | 2023 | PUBLIC, ENVIRONMENTAL & OCCUPATIONAL HEALTH | 2023 | 2024 | 5.68 | 2004 | chitosan hydrogels |
| 2023 | 2024 | 1.31 | 2023 | AGRONOMY | 2023 | 2024 | 5.56 | 2020 | oxidative stress |
| 2023 | 2024 | 1.18 | 2017 | BIOLOGY | 2022 | 2024 | 5.54 | 2022 | scaffold |
| 2023 | 2024 | 1.16 | 2023 | NUTRITION & DIETETICS | 2022 | 2024 | 5.36 | 2020 | silk fibroin |
| 2023 | 2024 | 1.06 | 2010 | AGRICULTURAL ENGINEERING | 2022 | 2024 | 5.23 | 2022 | generation |
| 2022 | 2024 | 0.93 | 2022 | ROBOTICS | 2023 | 2024 | 5.2 | 2019 | wound healing |
| 2022 | 2024 | 0.93 | 2022 | COMPUTER SCIENCE, ARTIFICIAL INTELLIGENCE | 2022 | 2024 | 5 | 2022 | vaccine |
| 2023 | 2024 | 0.71 | 2017 | MATERIALS SCIENCE, COATINGS & FILMS | 2022 | 2024 | 4.88 | 2018 | anticancer |
| 2022 | 2024 | 0.71 | 2015 | AUTOMATION & CONTROL SYSTEMS | 2022 | 2024 | 4.88 | 2019 | nanocomposite hydrogel |
| 2023 | 2024 | 0.65 | 2013 | PHYSICS, ATOMIC, MOLECULAR & CHEMICAL | 2022 | 2024 | 4.74 | 2017 | tumor microenvironment |
| 2022 | 2024 | 0.65 | 2011 | FOOD SCIENCE & TECHNOLOGY | 2023 | 2024 | 4.67 | 2023 | antioxidant activity |
| 2022 | 2024 | 0.65 | 2022 | AGRICULTURE, MULTIDISCIPLINARY | 2022 | 2024 | 4.62 | 2017 | nanocomposite |
| 2023 | 2024 | 0.58 | 2023 | PLANT SCIENCES | 2022 | 2024 | 4.61 | 2022 | t cells |
| 2023 | 2024 | 0.58 | 2023 | MATERIALS SCIENCE, CHARACTERIZATION & TESTING | 2022 | 2024 | 4.54 | 2022 | lipid nanoparticles |
| 2023 | 2024 | 0.58 | 2023 | ENGINEERING, MANUFACTURING | 2023 | 2024 | 4.49 | 2019 | optimization |
| 2023 | 2024 | 0.47 | 2006 | MICROBIOLOGY | 2020 | 2024 | 4.43 | 2016 | cancer immunotherapy |

Begin: the burst’ beginning year, End: the burst’ ending year, Strength: the burst’ strength index, Year: the first appearance time, Entity: the term.

Table S3. Summary of keyword clusters for the most recent stage(2019-2024).

| **ClusterID** | **Size** | **Silhouette** | **Average Year** | **Label (LLR)** | **Representative keywords** |
| --- | --- | --- | --- | --- | --- |
| 0 | 77 | 0.658 | 2020 | tissue engineering | drug delivery; tissue engineering; multifunctional injectable hydrogel system; cus nanoparticles; antibacterial treatment \| cancer therapy; ph-sensitive nanocomposite; antibacterial activity; alginate; scanning ion conductance microscopy |
| 1 | 76 | 0.561 | 2021 | immunogenic cell death | drug delivery; hydrogels; mechanisms; antioxidant activity; systems \| injectable hydrogel; cancer immunotherapy; postsurgical management; osteosarcoma chemo-immunotherapy; antioxidant activity |
| 2 | 71 | 0.631 | 2020 | nanocomposite hydrogel | drug delivery; tissue engineering; biomedical applications; synergistic treatment; multifunctional nanomaterials \| biomedical applications; mussel-inspired hydrogel; model; pedot synthesis; coaxial electrospinning |
| 3 | 61 | 0.645 | 2020 | skin cancer | drug delivery; polymeric drug delivery; macromolecular prodrugs; amino-functionalized mesoporous silica; carbon nanotube \| skin cancer; polymeric nanoparticles; wound healing; skin diseases; atopic dermatitis |
| 4 | 61 | 0.7 | 2019 | drug delivery | drug delivery; supramolecular polymers; adjuvant chemotherapy; carbon nanotube; carboxymethylated chitosan \| controlled release; nanoporous gamma-alumina; synergistic therapy; liver cancer; electrospun fibers |
| 5 | 58 | 0.71 | 2020 | cancer immunotherapy | drug delivery; iron oxide; in vivo; medicinal plant extracts; adjuvant chemotherapy \| drug delivery systems; colorectal cancer; local administration; local treatment; brain tumor |
| 6 | 56 | 0.718 | 2020 | graphene quantum dots | drug delivery; graphene quantum dots; ternary hybrid; mucoadhesive hydrogels; mucoadhesive microspheres \| controlled release; drug delivery system; cancer chemotherapy; solid dosage forms; site-specific delivery |
| 7 | 47 | 0.759 | 2020 | glioblastoma | drug delivery; peptide; tumor; brain; thermoresponsive hydrogels \| drug delivery system; alginate; temozolomide; adjuvant chemotherapy; vivo drug delivery |

Size: the number of articles in each cluster; Silhouette: the average contour value of clustering, it is generally believed that the clustering category with S > 0.5 is reasonable, and S > 0.7 means that the clustering is convincing; LLR: Log-likelihood ratio.

Table S4. The most trafficked keyword for the top five modules each year.

| **Year** | **2000** | **2001** | **2002** | **2003** | **2004** | **2005** | **2006** | **2007** | **2008** | **2009** |
| --- | --- | --- | --- | --- | --- | --- | --- | --- | --- | --- |
| Total modules | 7 | 6 | 4 | 6 | 7 | 6 | 9 | 9 | 11 | 9 |
| module1 | exploitation | solid_tumors | multidrug_resistance | tissue | chitosan_hydrogel | cervical_intraepithelial_neoplasia | biomedical_applications | antitumor_effect | affinity_chromatography_matrix | aqueous_solutions |
| module2 | hydrogel_nanoparticles | functionalized_derivatives | n_isopropylacrylamide | polymer_crosslinker | delivery_systems | copolymer | bacillus_calmette_guerin | tissue_adhesive | anti-tumor_efficacy | antitumor_activity |
| module3 | human_monocytes | regional_hyperthermia | hairless_mouse_skin | release | complex | chitosan_hydrogel | anti-cancer_drug | aggregation | 5_fluorouracil | angiogenesis |
| module4 | faecalis_var_myxogenes | - | excised_human_skin | n-_dimethylacrylamide) | ethylene_glycol)_nanospheres | colloid_nanoparticles | biodegradable_polymer | biodistribution | 2_methoxyestradiol | arterial_chemoembolization |
| module5 | colorectal_carcinoma | - | - | - | drug_delivery_systems | conjugate | bearing_1_aminolactose | basic_principles | adriamycin | tumor_targeted_delivery |
| **Year** | **2010** | **2011** | **2012** | **2013** | **2014** | **2015** | **2016** | **2017** | **2018** | **2019** |
| Total modules | 8 | 13 | 10 | 12 | 11 | 12 | 13 | 14 | 9 | 11 |
| module1 | ag-au_bimetallic_nanoparticle | tissue | prognostic_features | regeneration | diblock_copolymer | inclusion_complexation | beads | biological_activity | transport | 3d_printing |
| module2 | polymeric_micelles | 9l_gliosarcoma | mechanisms | prostate_cancer | formulations | functionalization | bevacizumab | controlled_drug_delivery | photodynamic_therapy | acrylic_acid |
| module3 | in_vitro_release | amphiphilic_graft_copolymer | polymeric_drug_delivery_systems | nanomedicine | extracellular_matrix | drug_release_behavior | cervical_cancer | beta_cyclodextrin | antibody | transdermal_delivery |
| module4 | hyperthermia | sensitive_hydrogels | management | photodynamic_therapy | drug_loading | gastric_cancer | anticancer_drug_delivery | transdermal_drug_delivery | antibacterial_activity | tumor_microenvironment |
| module5 | assembled_hydrogel_nanoparticles | triblock_copolymers | pharmacokinetics | intracellular_delivery | block_copolymer_micelles | co_delivery | cancer_immunotherapy | block_copolymer | adjuvant_temozolomide | agent |
| **Year** | 2020 | 2021 | 2022 | 2023 | 2024 |  |  |  |  |  |
| Total modules | 15 | 16 | 13 | 14 | 12 |  |  |  |  |  |
| module1 | silver_nanoparticles | brain_cancer | green_synthesis | hydrochloride | peritoneal_carcinomatosis |  |  |  |  |  |
| module2 | solid_tumors | epithelial_mesenchymal_transition | graphene_quantum_dots | antibacterial_activity | iron_oxide_nanoparticles |  |  |  |  |  |
| module3 | opportunity | alginate_hydrogels | combination_immunotherapy | hydrogel_beads | drug_delivery |  |  |  |  |  |
| module4 | polymerization | glucose | cross_linked_chitosan | antitumor_efficacy | release_kinetics |  |  |  |  |  |
| module5 | controlled_drug_delivery | block_copolymer_hydrogels | floating_hydrogel | zno_nanoparticles | carbon_dots |  |  |  |  |  |

Table S5. Summary of emerging topics.

| **ClusterID** | **Size** | **Silhouette** | **Average Year** | **Label (LLR)** | **Representative keywords** |
| --- | --- | --- | --- | --- | --- |
| 0 | 263 | 0.784 | 2019 | nanocomposite hydrogels | drug delivery; tissue engineering; 3d-printed scaffolds; antitumor; magnetic microrobot \| cancer therapy; tumor recurrence; postoperative treatment; in-situ drug delivery; residual tumor cell |
| 3 | 140 | 0.898 | 2018 | immunotherapy | drug delivery; cancer immunotherapy; lymph nodes; immune checkpoint inhibitor; injectable hydrogels \| cancer therapy; tumor recurrence; postoperative treatment; residual tumor cell; in-situ drug delivery |
| 8 | 61 | 0.99 | 2019 | quercetin | drug delivery; graphene quantum dots; ternary hybrid; double-emulsion system; fe3o4 nanoparticles \| carboxymethyl cellulose; controlled release; graphene oxide; cancer treatment; carbon nanotube |
| 10 | 39 | 0.937 | 2017 | pancreatic cancer | drug delivery; injectable hydrogels; stimuli-responsive materials; natural polymers; synthetic polymers \| pancreatic cancer; liposomal hydrogel; nir-ii photothermal therapy; carbon nanotube; n-isopropyl acrylamide |
| 18 | 14 | 0.98 | 2018 | oral cancer | drug delivery; graphene oxide; carboxymethyl cellulose; natural polymers; theranostic nanomedicines \| drug delivery system; raft polymerization; silica nanoparticle; molecular permeable compartments; biocatalytic reactors |
